# Transformative Insights into Community-Acquired Pressure Injuries Among the Elderly: A Big Data Analysis

**DOI:** 10.3390/healthcare13020153

**Published:** 2025-01-15

**Authors:** Sigal Shafran-Tikva, Gillie Gabay, Ilya Kagan

**Affiliations:** 1 Jerusalem College of Technology, Health Informatics, Givat Mordechai, Jerusalem 91160, Israel; 2 Research & Innovation Center, Hadassah University Medical Center, Jerusalem 91120, Israel; 3 Multi-Disciplinary Studies, Achva Academic College, Shikmim 79800, Israel; gillie.gabay@gmail.com; 4 Nursing Department, Ashkelon Academic College, Shikmim 78211, Israel; kagani@gmail.com

**Keywords:** big data, community-acquired pressure injuries, hospital, indicators, informatics, nursing clinical data, nursing homes

## Abstract

Purpose: To investigate community-acquired pressure injuries (CAPIs) in older people by utilizing big data. Design: Retrospective data curation and analysis of inpatient data from two general medical centers between 1 January 2016 and 31 December 2018. Methods: Nursing assessments from 44,449 electronic medical records of patients admitted to internal medicine departments were retrieved, organized, coded by data engineers, and analyzed by data scientists. Potential explanatory patient characteristics tested were gender, age, admission indices, nursing assessments including CAPIs, CAPI type and location, vital signs, and the results of lab tests within the first 36 h of admission. Findings: Most CAPIs were located in the buttocks (56.9%), followed by the sacrum (11.8%), ankle (10.8%), trochanter (5.1%), and leg (3.9%). Tissue associated with CAPIs was described as necrotic, serotic, bloody, granolithic, epithelial, and infected. There were 31% of first-degree CAPIs, 41% second-degree, and 18% third-degree. Previously unacknowledged patient characteristics associated with CAPIs are as follows: age, oxygen use, intestinal function, the touch senses of heat and pain, albumin, RDW (red cell distribution width), and systolic blood pressure. Conclusions: The novel indicators for CAPIs underscore the importance of data-driven approaches in detecting and preventing CAPIs in community care. These markers can detect and prevent pressure ulcers in the community, particularly among the elderly. Relevance for Clinical Practice: Nursing management is called upon to integrate information about novel patient characteristics associated with CAPI into clinical practice. Assimilating the insights from this hospital nursing-led study into community nursing will enhance the safety and quality of care for the elderly.

## 1. Introduction

The increase in life expectancy and the expected growth of the elderly population requires nurses to exploit data from various sources and healthcare facilities to improve the quality of care for the elderly in long-term care settings. Nursing informatics, which integrates nursing science with analytical science, can identify patients at risk and manage and communicate data in the evolving healthcare environment [1]. For example, employing predictive algorithms to identify high-risk situations can reduce readmissions and improve elderly patient outcomes [2]. The current study focuses on pressure injuries (PIs).

PIs are localized damage to the skin and/or underlying soft tissue due to intense and/or prolonged pressure, possibly in combination with shearing or from the use of a medical device. They are a frequent complication in patients with comorbidities and are associated with a higher risk of mortality [3]. Indeed, PIs are acknowledged to be one of the most significant signs of mistreatment and insufficient safety in the elderly [4,5]. Importantly, the associated morbidity, mortality, psychological distress, and vast annual expense due to hospital care may be preventable [6,7,8,9,10].

In this context, previous studies have long associated PIs with low-quality care and adverse health outcomes, especially among bedridden patients [5,11]. Several advisory panels have concluded that addressing PIs is a high priority [5,12].

PIs in the home or nursing home are a common consequence of lack of mobility support, insufficient methods of prevention, or poor understanding of skin breakdown and its consequences [5,13]. PIs on admission refer to PIs that are acquired in the community and are identified on hospitalization [14]. Such events are very common [5,12,15], with 77% of patients admitted to the hospitals presenting with community-acquired pressure injuries (CAPIs), even though only 21.4% were receiving homecare services for these PIs prior to their admission [4]. It should be noted that another study reported the prevalence of CAPIs as 7.4%, of which 76.1% were admitted from the community and 23.9% were admitted from long-term care institutions [16]. Still, other reports estimate the prevalence of CAPIs between 3.3% and 11.1% [4,17]. Accumulated data from long-term care, nursing homes, and rehabilitation facilities indicate a value ranging between 0.40 and 0.77 per 1000 adults [16,18]. Most CAPIs (58%) are superficial (Stage 1 or 2), 15% are deep-tissue PIs, and 22% are full-thickness PIs (Stage 3, 4, or unstageable). The most common anatomic locations for PIs are reported to be the ears (29%) and the feet (12%) [18].

A recent meta-analysis described the difficulty of prevention and treatment of PIs, whose continuous impact on clinical outcomes has a considerable cost [19]. This has led to growing efforts to prevent and treat PIs in hospitals. One study described a decision support model for the prevention of CAPIs in veterans with a spinal cord injury [20]. However, while nurses are responsible for the risk assessment of PIs in hospitals where they can use electronic health records to predict issues, the community lacks valid decision support tools for PIs, and there is a paucity of research into the prevention of PIs in the community [21,22]. Similarly, there is little information about CAPI-associated patient characteristics that could be useful for prevention [4,8,13,16,18,19,22,23,24].

A review of the last decade of literature indicates that the development of CAPIs is associated with a complex interplay of factors, but there remains a lack of understanding of the components and outcomes associated with effective care of CAPIs in the community [25]. Notably, most efforts to prevent PIs relate to events in acute care settings, and no study on CAPIs in the last decade has included socioeconomic factors [25]. There is, therefore, a consensus that developing comprehensive strategies to mitigate the occurrence and impact of CAPIs would be of great use [25]. This nurse-led research project used a big data analysis approach to examine the prevalence of CAPIs and identify predisposing characteristics among elderly patients admitted to hospitals from community nursing homes. To the best of our knowledge, this is the first study to use big data to identify risk factors for CAPIs based on hospital clinical data and nurse assessments.

## 2. Methods

### 2.1. Study Design

This was a retrospective study of elderly hospitalized adults discharged from internal medicine wards. Data were collected from electronic medical records from various departments in two general Israeli medical centers (900 and 350 beds) over 3 years (from 1 January 2016 through to 31 December 2018). The data were anonymized to satisfy regulations protecting patient privacy and to reduce the ethical challenges, but this made it impossible to analyze CAPI by socioeconomic status [26]. Patients with a comorbidity known to be associated with the indicated diagnosis (e.g., metastatic cancer) were excluded. The database of hospital records provided a diverse and large collection of mostly structured patient clinical data, including prior disease information, blood test results, descriptions of procedures, and patients’ assessment by nurses upon admission.

### 2.2. Study Variables

A dataset was created for this study using a predetermined code for PIs to identify patients with CAPIs at admission. Patients arrived at the medical centers from community nursing facilities or nursing homes. The presence of CAPIs was ascertained by the first skin assessment after hospitalization from the emergency department and/or within 36 h of admission to an internal medicine department. While the standard timeframe for CAPI identification is 24 h, preliminary analysis revealed no significant differences in the results after 24 or 36 h post-admission. Therefore, following expert consultation, the assessment window was extended to 36 h to reflect actual clinical practice while maintaining assessment validity.

Potential explanatory variables included the following: demographics (gender and age); clinical indicators (oxygen use, intestinal function, sensory impairments, e.g., heat and pain perception) and vital signs; laboratory results (albumin levels, red cell distribution width (RDW), and systolic blood pressure).

Patients readmitted within seven days of discharge (*n* = 2831, 6.3%) were excluded to avoid confounding hospital-acquired pressure injuries with CAPIs. This exclusion criterion was implemented because patients with recent hospitalizations have an altered risk profile due to their recent exposure to the hospital environment, and any pressure injuries identified upon readmission may have originated during their previous hospital stay rather than in the community setting.

Additionally, incomplete skin assessments (46.6% of records) were excluded. Comparisons of included and excluded cases showed no significant differences in demographic or clinical characteristics, suggesting random missingness. Due to the critical nature of the skin assessment data, imputation was not applied.

We avoided imputation because the skin assessment data were considered crucial to the primary outcome measure. Potential selection bias was examined by comparing the demographic and clinical characteristics (age, gender, and admission indicators) of the included and excluded cases.

### 2.3. Data Analysis

This study employed state-of-the-art big data analysis of patients with CAPIs upon admission to the hospitals and during their hospitalization in internal medicine, cardiology, hematology, and oncology departments. Data were retrieved and then organized and coded by data engineers to a data cloud dedicated to this study. They were then analyzed by data scientists to identify indicators for CAPIs that could be used for prevention and early treatment in the community, thereby avoiding hospitalization.

Categorical variables are presented by frequencies and percentages, with medians and interquartile ranges (IQR) used for continuous variables. Chi-squared tests were conducted on categorical variables, with Kruskal–Wallis tests used for continuous variables. Logistic regression was used to calculate the unadjusted odds ratio (OR), with a 95% confidence interval (CI) for CAPIs. A multivariate model for estimating the adjusted ORs of the clinical variables for CAPIs was assessed using logistic regression with a 95% CI. Data were collected and analyzed using Python software (version 3.6) and the Stats Models package (version 0.12.1). The categorical variables were recorded using dummy coding since one category for each was used as a reference category. An example is the binary categorical variables such as “mobility,” where 0 stands for “no impairment” and 1 implies “impairment.” For polytomous variables with greater than two categories, their levels are contrasted in the logistic regression model.

This ensured the proper inclusion and interpretation of these variables in the multivariate model.

Multivariate logistic regression was used to assess the relationship between the explanatory variables and CAPIs. Variables with clinical relevance and statistical significance in univariate analysis were included in the final model. Adjusted odds ratios (ORs) with 95% confidence intervals (CIs) quantified the strength of associations. The final model included pseudo-R^2^ (0.3244) as a measure of goodness-of-fit. Pseudo-R^2^ is used in logistic regression to assess the explanatory power of the model. While it is not directly comparable to R^2^ in linear regression, it provides a relative measure of how well the model explains the variability in the outcome. Analyses were performed using Python (version 3.6) and the StatsModels library (version 0.12.1).

## 3. Findings

A total of 44,495 medical records of hospitalized patients admitted to hospitals in the 3-year period were analyzed. We excluded 2831 (6.3%) for readmissions and 20,745 (46.6%) for lacking full skin assessments. In the process of admission to the emergency department, 2448 (5.5%) patients were diagnosed with CAPIs, of whom 1178 were female (48%) and 1270 male (52%), with an age range between 71 and 80 years. Nearly half (49.8%) of the patients were hospitalized for seven or more days. Table 1 presents the demographics, including gender, age, admission indicators, nursing assessments reported by nurses, vital signs, and the results of lab tests conducted within 36 h of admission.

The prevalence of CAPI was analyzed by considering the location, attributes, and degree/stage. Most CAPIs were located in the buttocks (56.9%), followed by the sacrum (11.8%), ankle (10.8%), trochanter (5.1%), and leg (3.9%). Tissue associated with CAPIs was described as necrotic, serotic, bloody, granolithic, epithelial, and infected. The most common grade of CAPIs (41%) was second-degree, with 31% rated as first-degree and 18% as third-degree. Table 2 presents the type, degree, and location of the observed CAPIs.

The results of regression analysis designed to identify indicators of CAPI are presented after adjustment for extreme and missing values. Most variables were categorical, except for individual continuous variables (e.g., age, number of CAPIs). Multivariate logistic regression was used to estimate the adjusted OR of the clinical variables for CAPIs. Table 3 presents variables that were significantly associated with CAPIs.

These results reveal significant variability in the effect sizes of different predictors. For example, mobility exhibited the highest adjusted OR (6.263), indicating a very strong association with CAPIs. Conversely, variables like age and systolic blood pressure had smaller adjusted ORs, suggesting weaker, though still significant, relationships. These findings emphasize the multifactorial nature of CAPIs and highlight actionable areas for intervention, such as improving mobility and monitoring albumin levels in at-risk patients.

## 4. Discussion

This study used big data to identify risk factors for CAPIs based on hospital clinical data and nurse assessments. The results of this big data study shed light on the characteristics and risks associated with CAPIs among elderly patients who arrived at the hospitals from nursing homes. The findings represent valuable insights that can be used to guide nursing practice in the community and inform future research and implementation strategies. Since CAPIs are often underreported in the community and there is consequently inadequate follow-up [27], our identification of new characteristics and risk factors associated with CAPIs provides an essential foundation for the development of preventive measures in community care [4,8,16,18,21,22,23,24]. A recent review of studies on CAPIs published over the last decade indicates that the development of PIs has been associated with a complex interplay of factors, although there remains a lack of understanding of the components associated with PI care in the community [25].

Risk factors previously considered to be associated with CAPIs include older age, impaired mobility, multiple comorbidities, and malnutrition [28,29]. A piezoelectric motion sensor, which provides a movement score based on the mean number of movements per hour, was used to assess patient mobility [30].

**Figure 1:**
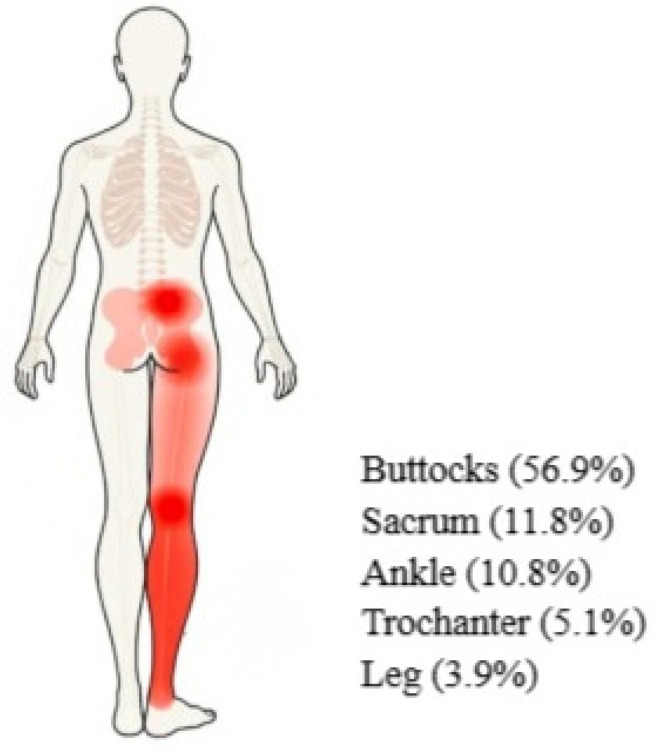
CAPI Distribution.

Interestingly, our findings identify polypharmacy as a key contributor to CAPIs. Polypharmacy is defined as the use of multiple medicines, which is a common practice in the older population and is associated with multimorbidity and adverse outcomes, including mortality, falls, adverse drug reactions, increased length of stay in hospital, and readmission to hospital soon after discharge [30]. Additional newly revealed contributing factors to CAPIs were poor albumin levels, RDW, systolic blood pressure, and poor intestinal function. Our results also suggest new locations for the development of CAPIs, namely in the buttocks (56.9%), sacrum (11.8%), ankle (10.8%), trochanter (5.1%), and leg (3.9%) (Figure 1). Tissue descriptions associated with CAPIs were necrotic, serotic, bloody, granolithic, epithelial, and infected. This new information can facilitate the ability of nurses to detect and manage PIs in the community.

Our results identify a strong hospital–community linkage, which introduces the potential for data-driven preventive measures and aligns with the principles of nursing informatics, where evidence-based practices are translated from data analysis to improve patient outcomes. Information about patients from nursing homes, gathered during hospital admissions, can enable community nurses to prevent morbidity and complications in elderly patients. The integration of data between hospital and community settings becomes ever more crucial, especially with the trend towards shorter hospitalizations and the provision of more care in the community.

Without targeted efforts to prevent CAPIs, there is a risk of repeated cycles of deterioration and readmission. Our results recommend informing clinical practice in community care based on big data analysis of high-quality evidence from hospital nurses who prioritize the prevention of CAPIs in the elderly. Community settings for the elderly have long been alerted to the need to provide safer care to patients through proactive diagnosis and treatment [30].

### 4.1. Managerial Implications

The insights from this study support those within the existing literature, such as the report by Friedman et al. [31] that elderly individuals with lower scores for daily activities had the highest rate of CAPIs. The vital role of nurses in preventive care and promoting quality of care makes an essential contribution to the economic and administrative aspects of community healthcare [19]. Nurses, armed with data they record and manage, can integrate effective preventive innovations, thereby enhancing patient safety and overall care quality [32]. The results of our study reinforce the pivotal role of nurses as the primary repository of patient knowledge and data, both in community settings and hospitals [33,34].

Previous studies have introduced various applications (Apps) for the prevention and treatment of PIs in acute care, which classify PIs through image processing on mobile devices [35]. The user uploads a photograph of the PI into the App, and the image is then processed to evaluate the probable stage of the PI based on an implemented algorithm, which then suggests cleaning procedures and provides the recommended treatment for the tissue type [36]. Since PIs are more common at home and in nursing homes where insufficient knowledge may hinder real-time care [4,13], we suggest that it may be useful to transform the informatics presented in this study into an AI-based App for community nursing care of PIs [1,37].

### 4.2. Study Limitation

The big data utilized in this study were obtained from two medical centers (900 and 350 beds, respectively) in Israel, which limits generalizations and calls for repetition. While these centers serve diverse populations and receive referrals from multiple community settings, we acknowledge that patterns of CAPIs may differ in other contexts, particularly in rural areas or smaller healthcare facilities with different resource levels and patient populations. In addition, the centers included in our study are major referral hospitals that may receive more complex cases and thereby affect the observed patterns and severity of CAPIs. Future multi-center studies incorporating a broader range of hospital types and geographical locations would be valuable to validate our findings across different healthcare settings and patient populations.

The exclusion of 46.6% of records due to incomplete skin assessments also represents a potential limitation. While our analysis of baseline characteristics suggests that the missing data was random, we cannot completely rule out selection bias. Future studies should emphasize complete documentation of skin assessments to minimize missing data and should consider employing multiple imputation methods when appropriate.

### 4.3. Conclusions

As health systems endeavor to enhance care quality while managing costs, addressing and preventing CAPIs becomes ever more imperative [23,38]. Nurse managers play a crucial role in promoting awareness of data-intensive analysis and knowledge-based nursing management in both hospital and community settings. The shifting landscape of patient care, with a trend towards shorter hospitalizations and increased community care, emphasizes the need to integrate information seamlessly between these environments. Our study advocates for the development of proactive measures to prevent CAPIs and encourages routine PI assessments in the community. The identification of new characteristics associated with CAPIs provides a foundation for targeted interventions. Nurse managers are encouraged to prioritize the integration of these characteristics into routine assessments, leveraging continuous data quantification for timely identification and prediction of PIs.

Our findings align with those of other studies on PIs in hospitals, thereby emphasizing the value of routinely collecting and assessing data [21]. This approach tasks community nurses with identifying patients at high risk of PIs and provides information for performance improvement. As the healthcare landscape evolves, the insights from this study underscore the pivotal role of data-driven strategies in preventing CAPIs, with the ultimate aim of enhancing patient care, minimizing complications, and optimizing resource utilization in both hospitals and the community [22,39].

## Figures and Tables

**Table 1:** Study population.

Sub Population	Events	%
Positive Skin Assessment (Within 36 h)	2448	5.5%
Negative Skin Assessment (Within 36 h)	18,471	41.5%
Readmission	2831	6.3%
Missing Skin Assessment	20,745	46.6%
Total Basic Population	44,495	

**Table 2:** Distribution of CAPIs based on assessment at admission (*n* = 2448).

Variable		Missing	* Number of Patients and %
Ulcer area		174	Less than 15 patients
	Trochanter		126 (5.1)
	Ear		Less than 15
	Abdomen		Less than 15
	Back		15 (0.6)
	Chest		Less than 15
	Arm		Less than 15
	Foot		19 (0.8)
	Shoulder		Less than 15
	Face		Less than 15
	Genitalia		Less than 15
	Sacrum		290 (11.8)
	Buttock		1393 (56.9)
	Spine		Less than 15
	Ankle		265 (10.8)
	Neck		Less than 15
	Leg		96 (3.9)
Degree of pressure injury	1	254	681 (31.0)
	2		901 (41.1)
	3		396 (18.0)
	4		216 (9.8)
Necrotic Tissue	No		2253 (92.0)
	Yes		195 (8.0)
Serotic Tissue	No		2222 (90.8)
	Yes		226 (9.2)
Bloody Tissue	No		2383 (97.3)
	Yes		65 (2.7)
Granolithic Tissue	No		2266 (92.6)
	Yes		182 (7.4)
Epithelial Tissue	No		1996 (81.5)
	Yes		452 (18.5)
Infected Tissue	No		2285 (93.3)
	Yes		163 (6.7)

* In big data analysis less than 15 is considered marginal and is not reported.

**Table 3:** Patient characteristics associated with CAPI on admission.

Characteristics	Adjusted *OR*	Adjusted *OR CI*	Adjusted *p*-Value	Unadjusted *OR*	Unadjusted *OR CI*
Age on admission	1.0102	[1.01, 1.01]	0.0000	1.039	[1.04, 1.04]
Multi-pharmacy	1.0132	[1.01, 1.02]	0.0001	1.0224	[1.02, 1.03]
Albumin level (lab)	0.9459	[0.94, 0.95]	0.0000	0.9167	[0.91, 0.92]
Red cell Distribution Width	1.0623	[1.04, 1.09]	0.0000	1.1141	[1.1, 1.13]
Systolic blood pressure	0.9952	[0.99, 1.0]	0.0008	0.9871	[0.99, 0.99]
Intestinal functions	1.9262	[1.62, 2.29]	0.0000	10.1404	[9.2, 11.17]
Eating habits	1.6759	[1.41, 1.99]	0.0000	9.0266	[8.23, 9.9]
Mobility	6.263	[5.0, 7.84]	0.0000	20.3565	[17.71, 23.4]
Conscious state	1.1814	[1.0, 1.39]	0.0477	6.6144	[5.97, 7.33]
Assessment of Senses	1.8584	[1.56, 2.21]	0.0000	3.6194	[3.23, 4.05]

Notes: Pseudo R^2^ = 0.3244; First skin assessment within 36 h from admission.

## Data Availability

Restrictions apply to the availability of these data. Data were obtained from Hadassah University Medical Center and are available from the authors with the permission of Hadassah University Medical Center.
